# Migratory Response of Cells in Neurogenic Niches to Neuronal Death: The Onset of Harmonic Repair?

**DOI:** 10.3390/ijms24076587

**Published:** 2023-04-01

**Authors:** Noelia Geribaldi-Doldán, Livia Carrascal, Patricia Pérez-García, José M. Oliva-Montero, Ricardo Pardillo-Díaz, Samuel Domínguez-García, Carlos Bernal-Utrera, Ricardo Gómez-Oliva, Sergio Martínez-Ortega, Cristina Verástegui, Pedro Nunez-Abades, Carmen Castro

**Affiliations:** 1 Departamento de Anatomía y Embriología Humanas, Facultad de Medicina, Universidad de Cádiz, 11003 Cádiz, Spain; 2 Instituto de Investigación e Innovación Biomédica de Cádiz (INiBICA), 11009 Cádiz, Spain; 3 Departamento de Fisiología, Facultad de Farmacia, Universidad de Sevilla, 41012 Sevilla, Spain; 4 Departamento de Biomedicina, Biotecnología y Salud Pública, Área de Fisiología, Facultad de Medicina, Universidad de Cádiz, 11003 Cádiz, Spain; 5 Department of Neuroscience, Karolinska Institutet, Biomedicum, 17177 Stockholm, Sweden; 6 Departamento de Fisioterapia, Facultad de Enfermería, Fisioterapia y Podología, Universidad de Sevilla, 41009 Sevilla, Spain

**Keywords:** neurogenesis, neuroblast migration, Alzheimer’s disease, Parkinson’s disease, Huntington’s disease, brain injury and stroke, epilepsy

## Abstract

Harmonic mechanisms orchestrate neurogenesis in the healthy brain within specific neurogenic niches, which generate neurons from neural stem cells as a homeostatic mechanism. These newly generated neurons integrate into existing neuronal circuits to participate in different brain tasks. Despite the mechanisms that protect the mammalian brain, this organ is susceptible to many different types of damage that result in the loss of neuronal tissue and therefore in alterations in the functionality of the affected regions. Nevertheless, the mammalian brain has developed mechanisms to respond to these injuries, potentiating its capacity to generate new neurons from neural stem cells and altering the homeostatic processes that occur in neurogenic niches. These alterations may lead to the generation of new neurons within the damaged brain regions. Notwithstanding, the activation of these repair mechanisms, regeneration of neuronal tissue within brain injuries does not naturally occur. In this review, we discuss how the different neurogenic niches respond to different types of brain injuries, focusing on the capacity of the progenitors generated in these niches to migrate to the injured regions and activate repair mechanisms. We conclude that the search for pharmacological drugs that stimulate the migration of newly generated neurons to brain injuries may result in the development of therapies to repair the damaged brain tissue.

## 1. Introduction

Pathologies that affect the central nervous system (CNS) and that involve neuronal death are the origin of important and burdensome neurological symptoms [1]. The search for the genetic, molecular, and environmental initiating factors that trigger neuronal death in not only neurodegenerative diseases [2,3,4,5] but also stroke and trauma [6,7] has received a great deal of attention within the last two decades with the goal of finding and designing new therapies. However, despite the efforts, no novel disease-modifying therapies have been proven significant at providing an effective benefit for patients with these devastating disorders. In this scenario, the discovery of new neurons being generated daily from neural stem cells (NSCs) within the adult mammalian brain has brought optimism for the treatment of these pathologies, and the search for therapeutic strategies based on stimulating neurogenesis in the adult brain has gained prominence over the past 10 years. Neurogenesis is the process by which new neurons are generated from neural stem cells (NSCs). NSCs are characterized by their capacity to divide both symmetrically—giving rise to other NSCs—and asymmetrically, producing new cells with a higher degree of differentiation but with the ability to generate a new progeny of neural cells [8,9]. This process has been extensively studied in the adult rodent brain, and it has been observed in the brains of other mammalian species. One of the pioneering studies in the field was the work of Gould et al. in 1999, in which the existence of newly generated cells with neuronal characteristics was described in the prefrontal, temporal, and parietal cortices of monkeys [10,11]. 

In order for neurogenesis to take place, the environment in which NSCs are located, known as the neurogenic niche, is almost as important as their own existence since the inflammatory molecules, growth factors, and matrix-bound signaling molecules condition NSCs fate towards mature functional neurons [12,13]. The neurogenic niche is a microenvironment characterized by a complex structure containing NSCs anchored to the basement membrane, soluble factors, extracellular matrix-bound molecules, and a high rate of vascularization [14,15]. Most of these soluble factors are produced locally and play a key role in the neurogenic process by favoring, among other roles, the constant production of new neurons [16]. In the adult brain, there are basically two neurogenic regions, which contain remnant populations of NSCs, that continue to produce neurons throughout life. These regions are the subventricular zone (SVZ), adjacent to the ependyma in the lateral walls of the lateral ventricles (LV), and the dentate gyrus (DG) of the hippocampus [15]. In the case of rodents SVZ, it has been well described that neuroblasts have the ability to migrate from the SVZ to the olfactory bulb (OB) through the rostral migratory stream (RMS) [17]. The microenvironment generated within the RMS allows neuroblasts to migrate tangentially from the SVZ to the OB. Once in the OB, neuroblasts change their migration pattern from tangential to radial and finally finish their differentiation process, becoming interneurons that integrate in the OB [18,19]. A recent in vivo study [20] demonstrates the existence of active NSCs within the OB core, meaning that the OB is another niche capable of generating neurons that then integrate into the preexisting circuits. New cells originating in the different niches participate in injury repair [21]. The DG of the hippocampus is another neurogenic niche, and it is involved in tasks such as memory and learning [22]. In the DG, neurogenic and proliferative activity is maintained in the adult mammalian brain, even in old individuals. Specifically, the sub granular zone (SGZ) contains astrocytes with radial glia characteristics, which can divide and form neural precursors or neuroblasts. These have the ability to proliferate and mature, giving rise to granular neurons that will settle in the granular layer [23], contributing to memory and learning processes [24]. The migration route in the SGZ is much shorter than in the SVZ, where neuroblasts need to cover a long distance after reaching the OB [25]. Other niches were described and are being studied, as in the case of the striatum, the neocortex or the hypothalamus [26,27,28,29]. 

As mentioned before, neurogenesis has been extensively studied in mammals, especially rodents, but also in humans. However, it has been described as having several differences between species. For example, striatal neurogenesis is only present in humans [30], while OB neurogenesis is present in other mammals but not in humans [31]. In this regard, adult human hippocampal neurogenesis (AHN) is now in the spotlight because of the high level of controversy surrounding the aging process. In nonhuman primates and mice, it was reported a clear decline of AHN neurogenesis [32,33] through aging. However, Boldrini et al. used healthy human autopsy samples of the hippocampus and observed a similar number of intermediate neural progenitors and immature neurons in the DG of individuals at different stages of life (from 14 to 79 years old) [34]. Simultaneously, Sorrells et al. described a sharp decline in the production of new neurons in the human DG during the first year of life, becoming nearly nonexistent after the first year [35]. These apparently contradictory results aroused great controversy, and authors including Flor-García et al. identified the problem in how the human post-mortem samples were handled and provided an alternative and promising protocol to better control immunohistochemistry [36]. 

An association of alterations in neurogenesis in both niches with cognitive impairment has been described in mouse models of metabolic alterations [37,38,39], cerebrovascular damage [40], aging [41], and neurological disorders [42,43] and in general, increased organized, non-aberrant neurogenesis leads to improved cognitive performance. In addition to the generation of neuroblasts and their maturation, post-neurogenic migration is another mechanism that undergoes significant changes during different disease processes. However, more extensive studies are required to properly understand the migration mechanisms and their role in different neurological disorders. Neurodegenerative diseases such as Parkinson’s disease (PD), Alzheimer’s disease (AD), or Huntington’s disease (HD) have a deep impact on brain configuration, even modifying the anatomical structure and its associated physiological conditions. These structural modifications could be observed also in stroke and trauma. The neuronal loss generates distinctive consequences, including decreased cognitive function, impaired attention, and even personality alterations [16,44]. In order to find strategies to compensate this neuronal loss, researchers worldwide have contributed to the understanding of the neurogenic processes, including migration, as well as the intrinsic characteristics of the neurogenic niches, which play a key role in neurogenesis as they harbor the NSCs from which new neurons derive.

In this review, we analyze how the neurogenic niches respond to different pathologies, focusing on the capacity of their progenitors to migrate to the structures involved in these diseases through the activation of repairing mechanisms.

## 2. Neurogenic Niches and Migration

The SVZ and the DG of the hippocampus are the two most studied neurogenic niches in the adult brain. These niches have a particular histological configuration that provides the required conditions for NSC survival, differentiation, and later stimulation of migration to their final destinations. The human SVZ is composed of four layers: (i) the ependymal layer, a monolayer that contacts the lateral ventricle; (ii) the hypocellular layer; (iii) the astrocytic ribbon; (iv) and the transitional layer. NSCs are located in the astrocytic ribbon, surrounded by ependymal cells, astrocytes, oligodendrocytes, and endothelial cells [45]. The SVZ was extensively studied in rodents, and different cell types have been described as follows: astrocyte-like NSCs, also called type B1 cells, share features with other niche astrocytes, including an apical process that contacts the cerebrospinal fluid (CSF) contained within the LV. These B1 cells give rise to the B2 cells, which have no contact with the LV but maintain their astroglial characteristics. B1 cells also generate type C cells, sometimes referred to as transient-amplifying C cells, that have the ability to divide quicker than B1-type cells and to differentiate into neuroblasts (type A cells) [9]. The tendency of NSC within this niche is to remain quiescent. The balance between the quiescence of NSCs and their proliferation is crucial for adult neurogenesis. In fact, most B1-type cells are quiescent, and only in the presence of specific external stimuli they are activated in order to generate new neurons [46]. Several signals trigger the activation of NSCs, such as insulin and other factors released by glial and endothelial cells within the SVZ (reviewed in [47]).

In the DG neurogenic niche, NSCs are located in the SGZ of the DG and show different stages of differentiation. Type 1 cells (or radial glia-like neural stem cells) make contact with blood vessels from the hilus through a cilium and to the granular zone and the molecular layer through one long process and branches, respectively. These cells are multipotent and have the capacity to remain quiescent for long periods of time [48]. By asymmetrical divisions, NSCs originate as type 2 cells (or non-radial transit-amplifying cells), which are subdivided into type 2a cells (glial-like type) or type 2b cells (doublecortin positive, or DCX+, cells). Type 3 cells show a neuroblast phenotype and migrate to the granule cell layer [49]. 

The migration process of newly generated neuroblasts is not only important in the early stages of development but also in the postnatal brain, the adult healthy brain, and brain pathologies. The regulation of migration is essential in the adult brain for maintaining and completing the neuronal circuitry. In this sense, the milieu of chemical molecules, physical scaffolding, and cellular interaction that governs the fine control of neuroblast migration has been extensively studied over the years, focusing especially on the physiological migration through the RMS and pathophysiological migration toward infarcted areas of the brain (reviewed in Young et al., 2021) [50,51,52]. Neuroblasts originated in the SVZ exhibit saltatory movements during their migration, which are closely related to their bipolar morphology [53,54,55]. This morphology is characterized by the existence of a long leading process and a trailing process that are essential for the individual movement of neuroblasts and occur as follows: first, cell microtubule modifications and (F)-actin accumulation in the leading process are involved during cell extension; then, a swelling formation and a centrosomal migration take place to produce a somal translocation [54,55,56]. Moreover, these migrating neuroblasts form chains both within the RMS and when migrating towards injured areas that allow cells to use each other to migrate [57]. In the case of trauma and stroke, neuroblasts are able to migrate toward the injured site not only in chains but also individually, following bidirectional movements [58]. Trying to reach the damaged area, neuroblasts also migrate along newly-generated blood vessels (a process that is not yet fully understood) [59], astrocytic processes (typical in RMS migration) [57], and along the radial glia, which are common in the developing brain [50,60]. Molecules such as the matrix metalloproteinases (MMPs) are important for the extracellular matrix remodeling that facilitates neuroblast migration toward the injured area [61,62]. The clustered chains are based on adherent junctions and binding proteins such as laminin and integrins [59,63,64], while their organization is influenced by molecules such as neuregulin 1 [65,66] (Table 1). Neuroblasts also physically interact with other types of cells within the RMS, such as astrocytes [67] and endothelial cells from blood vessels, which act as scaffolds [68,69,70]. Several molecules are implied in the interactions between neuroblasts and these scaffolds, both in physiological [67,68,71,72] and pathophysiological [73,74,75,76,77] migration processes, as summarized in Table 1. Remarkably, the migration patterns—directionality, velocity, and even the pathway followed by neuroblast—are determined by several soluble factors diversely distributed throughout the SVZ, RMS, and OB, and even in the injured areas (Table 1). Thus, different chemoattractant molecules have been described within the OB [78,79,80,81,82] and injuries [83,84], while chemo-repulsive molecules such as Slit1, Slit2 [85,86], and insulin growth factor 1 (IGF-1) [87] have been detected in the LV and SVZ, fostering the entry of SVZ neuroblast into the RMS and their migration toward the OB. Interestingly, other molecules have been described as playing an even more complex role in the regulation of migration, as is the case with GABA [88]. In 2004, Bolteus and Bordey published that GABA secreted by neuroblasts reduced the migration rate via activation of GABA_A_R within these neuroblasts. According to this study, GABA concentration was modulated by surrounding astrocytes, which expressed the GABA transporter GAT4, thus modulating undirectedly the velocity of migration [88]. However, a more recent study argued that neuroblast-secreted GABA triggered the expression of TrkB—a high-affinity brain-derived neurotrophic factor (BDNF) transporter—in the membrane of astrocytes, therefore trapping BDNF from the medium and promoting the entry of migrating neuroblasts into a stationary phase [68]. Other interesting molecules that participate in neuroblast migration are neuregulins (NRG); NRG2 infusion in the SVZ leads to a higher number of neuroblasts emigrating from the SVZ toward the OB. On the contrary, infusion of NRG1 in the SVZ impairs OB emigration. This impairment is explained by the role of NRG1 as a chemoattractant. The introduction of a NRG1-expressing source in an area adjacent to migrating neuroblasts in the RMS directs these cells towards the ectopic source, indicating that a NRG1 source is able to attract migrating neuroblasts [66].

## 3. Alzheimer’s Disease

**Figure 1:**
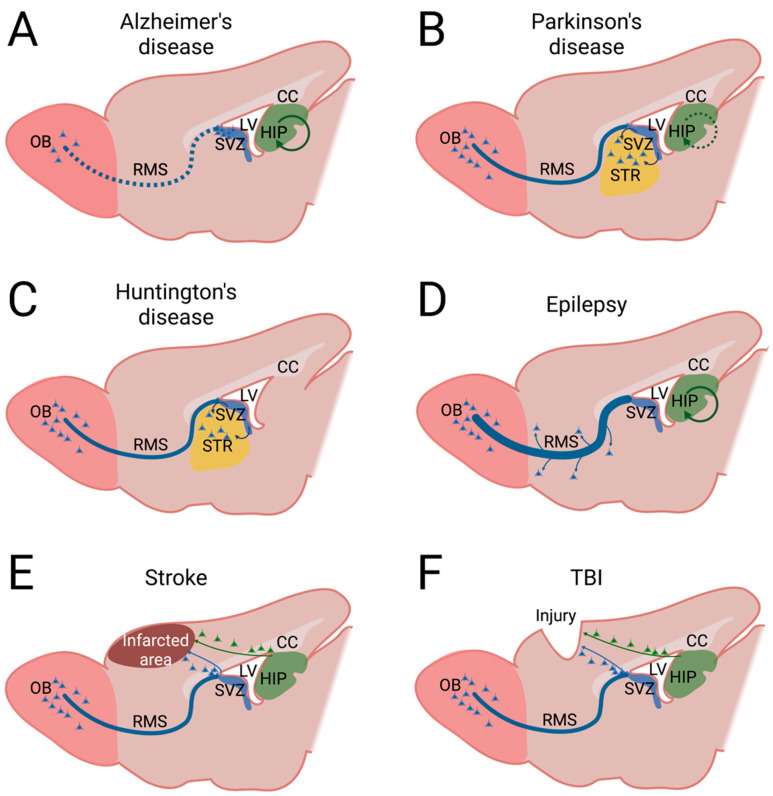
Changes in cell migration in different diseases/injuries. Sagittal view of brains affected by different diseases/injuries and the areas in which migration is altered. Normal arrows indicate the origin and final destination of aberrantly migrating cells; circled arrows represent changes in migration within the same area; dashed lines represent a decreased rate of migration; a thicker line in the RMS (epilepsy) represents an increase in cell migration velocity compared with normal migration within the RMS (Parkinson’s disease, Huntington’s disease, stroke, and TBI). (**A**) Alzheimer’s disease. It has been described as undergoing regional changes within the hippocampus and a detriment to the velocity of migration along the rostral migratory stream, with accumulation of neuroblast in the subventricular zone and fewer neurons in the olfactory bulb. (**B**) Parkinson’s disease. The figure depicts aberrant migration of neuroblast from the subventricular zone to the striatum and retarded migration within the hippocampus. (**C**) Huntington’s disease. As the picture shows, it has been confirmed a clear aberrant migration of neuroblasts from the subventricular zone towards the striatum. (**D**) Epilepsy. As depicted, in different forms of epilepsy, it has been described as aberrant migration within the hippocampus, whereas a higher migration velocity rate has been demonstrated toward the rostral migratory stream, with ectopic migration to non-olfactory areas. (**E**) Stroke. As it can be seen in the figure, after a stroke, the new neuroblasts formed in the subventricular zone and the hippocampus can potentially migrate to the infarcted area. (**F**) Traumatic brain injury (TBI). As represented by the figure, it has been shown that both the subventricular zone and the hippocampus react to the injury, and the new neuroblasts alter their migration patterns, trying to reach the lesion. CC: corpus callosum; HIP: hippocampus; LV: lateral ventricle; OB: olfactory bulb; RMS: rostral migratory stream; STR: striatum; SVZ: subventricular zone; TBI: traumatic brain injury. Created with BioRender.

AD is a neurodegenerative disease that causes progressive neuronal loss and is the most common cause of dementia in the elderly population. Clinically, it is characterized by a progressive decline in cognitive abilities and memory loss, accompanied by behavioral abnormalities. It is estimated that the number of people suffering from AD or other dementias will increase from the 57 million cases worldwide detected in 2019 to more than 152 million cases in 2050, with a female-to-male ratio of 1.69 [92]. Although sex differences can be partially explained by a longer life expectancy in women, alternative sex-driven differences in the biological mechanisms underlying AD have been suggested as well, such as a greater association between Apolipoprotein E and CSF tau levels in women, especially in amyloid-positive patients [93]. Histopathology of AD has been well studied and is represented by neuronal loss in the cerebral cortex, the hippocampus, and the amygdala; it is also associated with the presence of senile plaques containing β-amyloid peptide (Aβ) and neurofibrillary tangles (NFTs), which are composed of straight or helical filaments of abnormally hyperphosphorylated tau [94]. The highest concentration of NFTs is located in the hippocampus and in the entorhinal cortex (EC). However, in the early stage of the disease and even before the onset of significant cognitive impairment, amyloid deposition predominates, whereas neuronal and synapse loss often leads to NFT formation, thus AD progression and cognitive decline can be correlated with NFT pathology [95]. To understand the pathology associated with AD, it is necessary to describe the characteristics of the regions involved and their connectivity. Connections in the hippocampus occur through a tri-synaptic circuit, in which the pyramidal cells located in layer II of the EC connect to the cells of the DG, which in turn connect to the CA3 area of Cornu Ammonis. The signal is then projected to the pyramidal neurons in the CA1 area, and from there it is sent back to the EC [96]. In the human hippocampus, a brain area essential for learning and memory, neurogenesis occurs throughout both physiological and pathological aging conditions, but it has been shown that neurogenesis is reduced in the hippocampus of patients with AD [43,97]. The neurogenic decline worsens cognitive deficits, and it is often a prelude to the characteristic symptoms of AD. Thus, it has been proposed that adult neurogenesis is a potentially relevant mechanism underlying cognitive impairment in AD patients [43]. Studies related to AD and neurogenesis are in the spotlight of research, being the field of investigation for many groups worldwide. Some studies revealed a high expression of neurogenic biomarkers such as DCX, PSA-NCAM, NeuroD, or Calbidina [98]. In contrast, postmortem studies demonstrated changes in the proliferation markers Ki67 and nestin, while the DCX and the neuronal marker βIIItubulin remained unchanged. These results suggest that, although the number of NSCs increases, this change is not reflected in an increase in the number of generated neuroblasts [99]. Tobins et al. demonstrated regional changes in the hippocampus of brains with AD (Figure 1A). They show that nestin+ cells are located in the ventral hippocampus, while nestin+/Sox2+ and PCNA+/DCX+ cells are more evenly distributed along the dorsal/ventral axis of the hippocampus [97]. This phenomenon could be associated with the function of the different areas of the hippocampus, with the dorsal area implicated in learning and memory and the ventral hippocampus associated with emotions and stress response [100,101]. Recently, Márquez-Valadez et al. pointed out that in AD, newly generated dentate granule cells within the granular layer of the hippocampus show morphological alterations such as dendritic atrophy that contribute to the progression of the disease [102]. For the purpose of elucidating the mechanisms responsible for the alterations in hippocampal neurogenesis observed in AD, several murine models—including transgenic mice—have been used. While some studies attribute the deficiency in neurogenesis to the accumulation of Aβ [103,104], others describe a decrease in BrdU+ cells and DCX+ cells in the SGZ accompanied by an abnormal maturation of the newly generated neurons [105]. 

Related to the migration of newborn neurons, research with tau knockout mice demonstrates that tau is not really necessary for neuronal survival while it is crucial to the migration and integration of these neurons into the neuronal circuits [106]. Pallas-Bazarra et al. revealed that DCX co-localizes with phosphorylated tau at epitopes that are usually markers for pathological tau in the later stages of AD [107]. Esteve et al. in 2021 demonstrated that migrating cells accumulate in the SVZ of APP/PS1 mice. This research group found an increase in Cdh1 levels and a decrease in Cdk5/p35 and cyclin B1, which is evidence that these cells exhibit an alteration of the cell cycle. Furthermore, they found fewer cells in the RMS and fewer OB neurons, according to the deficit in cell migration and the decrease in odor discrimination (Figure 1A) [108]. 

## 4. Parkinson’s Disease

PD is the second most common disorder associated with neurodegeneration, and it is characterized by a progressive motor disability [109,110]. This terrible disease affects more than 1% of the elderly population [111], and it is produced by the loss of dopaminergic neurons within the substantia nigra pars compacta located in the mesencephalon. This loss of neurons disrupts the communication with the striatum and contributes to the aggregation of α-synuclein (α-syn), causing the formation of Lewy bodies and the triggering of an astroglial reaction [112]. At the same time that these dopaminergic neurons degenerate, the compensatory mechanisms fail [113,114]. Regarding the causes that could underlie the pathogenesis of PD, up to 10% of the PD cases have genetic implications, with aging still representing the most crucial event in PD development [111,113]. In fact, most of the pathophysiological changes are due to the aging process and are also linked to inflammation and oxidative stress [115,116].

Pathophysiological causes of α-syn accumulation are still under discussion, but some of these determining factors have already been identified: genetic alterations, oxidative stress, neuroinflammation, mitochondrial alterations, and aberrant neurogenesis [117,118,119,120,121].

Regarding neurogenesis in PD, Ernest et al. in 2014 argued that NSCs originated in the human SVZ may generate neuroblasts that are able to migrate to the striatum (Figure 1B) [28]. In additon, the possible existence of neurogenesis in the substantia nigra is currently under debate [122,123,124]. Moreover, some authors have described the presence of neural progenitors in the tegmental aqueduct periventricular region, near the substantia nigra pars compacta [125,126]. Recently in 2021, Bender et al. uncovered an increase in early-stage Pax6-expressing progenitors in the SGZ of the hippocampus in a mouse model of α-syn pathology, as well as an increase in the number of reactive astroglia in other regions of the hippocampus [127]. Supporting these data, Terreros-Roncal et al. found that patients with α-synucleopathies show an increased number of NSCs, proliferative neuroblasts, and new granular neurons. They also observed that these alterations are more pronounced in patients with PD than in those with dementia with Lewy bodies [42]. The increase in neurogenesis may be triggered as a repair mechanism trying to compensate for the neuronal loss that occurs in PD, although dysfunctionalities in this and other mechanisms [42] would not be enough to stop the progression of the disease. Contrary to the studies discussed above, other authors pointed out that neurogenesis may be reduced in PD. Thus, it has been suggested that neurogenesis decreases in the SVZ and SGZ of a 6-hydroxidopamine model of PD as a consequence of the loss of dopaminergic and noradrenergic stimuli derived from neuronal loss [123,128]. Nonetheless, other authors have provided conflicting results using the same animal model, arguing that dopaminergic and noradrenergic stimuli do not affect proliferation [129] or even that proliferation is stimulated in PD, promoting astrogliosis without affecting neurogenesis [130]. In genetic forms of PD, deficiency of molecules such as PINK1 or parkin has also been shown to disrupt neurogenesis in the SGZ and the SVZ [131,132]. Moreover, the Wnt/β-catening signaling cascade is one of the most studied pathways in aging and neurodegenerative diseases, including PD [133,134,135,136]. L’Episcopo et al. described that this pathway is downregulated in aging (implicating oxidative stress and inflammation) within the neurogenic niches while Wnt-antagonists are upregulated. These changes may seriously impair SVZ neurogenesis, NSC activation in the tegmental aqueduct and periventricular region, and the repair of dopaminergic neurons [137,138,139].

Most PD studies related to migration refer to resident NSCs in the SVZ and their relationship with the striatum due to its closeness [140,141]. However, Jiang et al. reported a decrease in hippocampal NSCs in a mutant mouse model of PD. As a consequence, they observed a retarded migration (Figure 1B) of differentiated cells, which evidenced non-motor symptoms arising from this neurogenesis impairment [142]. 

More in-depth studies are necessary to elucidate the relationship between neurogenesis and PD, which is a current focus for researchers around the globe. 

## 5. Huntington’s Disease

HD is a devastating, monogenetic neurodegenerative condition. Typically, symptoms start to become evident in the 3rd or 4th decade of life, with uninterrupted progression thereafter. It is caused by a CAG trinucleotide repeated expansion within the huntingtin gene (HD/IT15) [143] and is translated into polyglutamine (polyQ) stretches in the huntingtin protein [144,145] causing misfolding and aggregation [146,147] and resulting in the disease’s development. HD is characterized by a specific degeneration of medium spiny neurons in the striatum and a low turnover rate of neuroblast [28,148]. As a result, in the striatum of HD patients, it has been described as having neurotransmitter dysfunction, oxidative stress, microglial activation, and reactive astrogliosis, factors identified as second-degree clinical effects [149]. The prevalence of HD is estimated at 10 cases per 100,000 inhabitants. These rates are higher in North America, northwestern Europe, the Middle East, and Australia—where estimates range from 5.96 to 13.70 cases per 100,000 inhabitants—than in Asia, where rates are estimated to range from 0.41 to 0.70 cases per 100,000 inhabitants [150]. 

The current available data regarding the effect of HD on cell proliferation are highly controversial. Thus, while one study revealed an enhanced cell proliferation rate in the subependymal layer of the adult human brain [151], other authors did not observe any change in proliferation in the SVZ when studying R6/2 mice [152] or even showed a decrease in BrdU positive cells in the SVZ using a tgHD rat model, as in the case of the study of Kandasamy et al. [153]. However, these two authors confirm a clear migration of DCX^+^ neuroblasts from the SVZ towards the striatum (Figure 1C). In 2014, Ernest et al. conducted a study in human brains, measuring nuclear bomb test-derived ^14^C on the DNA of proliferating cells in order to perform a retrospective analysis of cell replacement, specifically focusing on neuroblasts and neurons in the striatum of individuals at various stages of HD. The study demonstrates that striatal neurons in HD patients have considerably lower turnover rates compared with nonaffected age-matched persons [28]. Focusing on the hippocampus, studies in different animal models revealed a decrease in the proliferation and survival of NPCs [154,155,156,157]. Stanley and colleagues observed that R6/1 mice had a lower number of DCX+ cells in the DG. Moreover, Gil et al. found that the total number of BrdU+⁄NeuN+ cells was 76% less in the R6/2 compared with controls [157]. In a deeper study, Aigner and Bogdahn revealed an increase in the transforming growth factor (TGF)-β signaling in HD, which is consistent with several studies related to neurodegenerative diseases. Specifically, TGF-β1 appears to be a key modulator of neurogenesis as it triggers NSC quiescence through the presence of pSmad2, a downstream target of TGF-β signaling observed in Sox2+/GFAP+ SGZ cells [158]. As the proliferation rate of NSC was reduced, an increase in neuroblast proliferation was induced as a compensatory mechanism [154].

A clear neurogenic response is found in the SVZ and DG of murine models of HD and in human patients, which requires further studies aimed at elucidating the role that neurogenesis and migration play in the development of this disorder.

## 6. Epilepsy and Adult Neurogenesis

Epilepsy is a group of mixed neurological disorders whose common core feature is spontaneous, recurrent, and unpredictable seizures. Several studies have highlighted how seizures have various molecular, cellular, and physiological consequences, especially in the developing brain but also in adults. Thus, abnormal neurogenesis, migration (Figure 1D), and neuronal cell loss are hallmarks of cellular responses to prolonged seizure activity [159]. However, the overall effect of a seizure on neurogenesis depends on the type of seizure [160]. For example, acute seizures increase hippocampal neurogenesis and cause changes such as abnormal integration of hilar basal dendrites, hilar ectopic migration, mossy fiber sprouting in DG-generated cells, and abnormal migration of newborn neurons into the DG [160,161,162,163,164,165]. Thus, in rodent models of epilepsy, the generation of new neurons in the granule cell layer of the DG is increased following pilocarpine-induced status epilepticus [162] or kindling stimulation [166]. Furthermore, increased proliferation of NSCs in the SGZ was observed shortly after the status epilepticus, resulting in increased production of new neurons following excitatory stimulus-mediated seizures [167,168,169]. The prompt proliferative response appears to be mediated by radial glial type 1 cells, whereas at the peak of cell proliferation, activation of DCX-expressing neuroblasts is increased [167,168,170]. Examination of the hippocampus of young patients with temporal lobe epilepsy also showed increased cell proliferation and neural progenitors, supporting findings in animal models [171]. As previously described, increased hippocampal neurogenesis after acute seizures is associated with abnormal migration of granule cells into the hippocampal circuit [162,172,173,174,175]. For example, Scharfman et al. showed that after pilocarpine-induced status epilepticus, a population of nascent granule neurons migrated out of the granule cell layer, and some neurons reached the hilar/CA3 junction, where they were abnormally integrated into the CA3 network [176]. This finding is also supported by other groups who have found abnormal integration and hyperexcitation of hilar ectopic granule-like neurons after acute seizures [162,174,177,178,179]. Ectopic granule cells have also been found in the tissues of epileptic patients. The mechanisms underlying the migration of seizure-derived granule cells into the hilus remain unclear. It has been proposed that seizures induce abnormal chain migration of granule cell progenitors toward the hilus [180] and this aberrant migratory behavior may be initiated by the loss of reelin signaling [174,181]. Furthermore, mTOR signaling was recently shown to be associated with the migratory behavior of newborn granule cells, and overactivation of the mTOR signaling pathway led to abnormal migration of newborn cells in the hilus, a phenotype that replicated some of the effects of seizures on DG neurogenesis [182].

Sustained epileptic activity also resulted in a dramatic increase in cell proliferation in the subventricular zone. Neuroblasts generated in response to seizures migrated more rapidly to the OB, and a fraction left the RMS prematurely to invade non-olfactory forebrain regions (Figure 1D). Neuroblasts that reach the cortex rarely seem to survive [183,184]. Seizure-induced neurogenesis has been reported to exert a pro-epileptic effect that contributes to the progression of initial seizure-induced hippocampal damage to chronic epilepsy [185]. Several results support this hypothesis: (i) ectopic granule cells appear to be abnormally synchronized with spontaneous, rhythmic bursts of CA3 pyramidal neurons [17,176]; (ii) a portion of seizure-generated granule cells have, in addition to their normal apical dendrite, an additional basal dendrite that extends toward the hilus and receives excitatory input from mossy fibers and could, thus, form a recurrent excitatory circuit [177,178]; (iii) aberrant neurogenesis is sufficient to induce spontaneous seizures in an otherwise intact animal [182]; (iv) silencing aberrant granule cells reduces epilepsy pathology and seizures [161,186,187]; (v) sustained inhibition of neurogenesis after seizures induced a transient suppression of spontaneous seizures [188].

While epilepsy is associated with increased neurogenesis and abnormal integration of new cells early in the disease, neurogenesis is preserved or reduced in chronic epilepsy, as the overall reduction in neurogenesis was significantly greater in rats with increased numbers of spontaneous seizures [189,190,191]. Furthermore, alteration in neuronal differentiation, with increased production of new astrocytes, has also been found in chronic epilepsy [164,192,193,194]. Existing analyses suggest that reduced neurogenesis is the result of a marked reduction in the neuronal differentiation of newborn cells rather than reduced new cell generation or a marked reduction in the number of neural stem cells [164,195,196,197]. Although, in a mouse model of Dravet syndrome—an encephalopathy with severe epilepsy that occurs during infancy—a marked decrease in NSCs was found in addition to impaired cell division and aberrant neurogenesis [198]. Impaired neuronal differentiation of newly generated cells in chronic epilepsy appears to be related to the presence of an unfavorable microenvironment in the hippocampus, characterized by reduced concentrations of several factors that promote neurogenesis. According to the proposed function of hippocampal neurogenesis, a marked decrease in DG neurogenesis may contribute to the persistence of seizures, learning and memory impairment, and depression seen in chronic epilepsy [160].

## 7. Neurogenesis in Stroke

A stroke is a serious medical emergency that occurs when blood flow to the brain is interrupted. This can be due to a vessel blockage (ischemic stroke, which occurs in over 80% of cases) or a hemorrhage (hemorrhagic stroke). The lack of oxygen and glucose caused by the loss of blood flow leads to the death of neurons and results in an acute loss of function in the area of the brain supplied by the affected vessel. The initial cell death is followed by a longer structural and functional reorganization period. Stroke can cause symptoms such as weakness, difficulty speaking, sensory deficits, and difficulty walking [199,200]. Stroke represents the second leading cause of death and the third leading cause of disability globally [201].

As we mentioned before, the CNS has a limited capacity for repair, as demonstrated by previous research [202]. However, it has been observed that a certain degree of spontaneous recovery from brain ischemia can occur [203]. Several mechanisms, such as neurogenesis, angiogenesis, axonal sprouting, and synaptogenesis, mediate this recovery process [204,205]. Experimental evidence suggests that functional improvement following stroke may be mediated by the replacement of lost neurons by activating endogenous NSCs [206]. The proliferation of NSCs and their progeny in the adult brain following stroke has been demonstrated in both human and animal models [207]. In animal models, stroke has been found to stimulate the proliferation of NSCs in the SVZ and the SGZ of the DG. These progenitor cells can potentially migrate to the affected area and differentiate into neurons, contributing to improved functional outcomes post-stroke (Figure 1E) [208,209,210]. This migration begins 3–4 days after stroke and is able to continue for at least 4 months, where reactive astrocytes, activated microglia/macrophages, blood vessels, and migratory scaffolds formed by astrocytic processes are essential for the movement of neuroblasts toward the injured site. In fact, after a stroke, there is an increase in the density of blood vessels, which increases blood supply to the injured brain regions. Endothelial cells secrete growth factors such as BDNF and angiopoietin that promote neuroblast recruitment within the lesion area, promoting proliferation and migration from the SVZ. The molecular and migratory capacities of SVZ neuroblasts are altered in stroke; genes such as Hif-1α or Notch4 are upregulated, and molecules such as MMPs are overexpressed in SVZ neuroblasts in response to stroke in order to facilitate emigration [50].

The timeline of the neurogenic process after stroke varies depending on the location and severity of the stroke, as well as the species studied. In general, studies have found that neurogenesis begins within a few days after the stroke and can continue for several weeks [206]. The in vivo studies performed by Zhang et al. have shown that following a stroke, there is a significant increase in the proportion of dividing cells within the SVZ. This increase starts at 2 days post-stroke (24%), peaks at 7 days post-stroke (31%), and at 14 days post-stroke, the proportion of dividing cells returns to the baseline level observed at 2 days post-stroke. Additionally, the cell cycle length of SVZ cells undergoes dynamic changes over a period of 2–14 days following a stroke. The shortest cell cycle length observed was 11 h at 2 days post-stroke, which is significantly shorter than the cell cycle length of 19 h in non-stroke SVZ cells. There is a gradual increase in the cell cycle length by 4 days post-stroke, and by 14 days post-stroke, it reaches the length of non-ischemic progenitor cells [211,212,213]. Studies in human subjects have demonstrated that the extent of neurogenesis following a stroke is less extensive than in animal models. Jin et al. found an increase in the number of new neurons in the hippocampus during the initial week post-stroke; however, this number returned to pre-stroke levels within 2–3 months [98]. It is important to note that while neurogenesis has been observed in certain brain regions following a stroke, the extent and duration of this process are still an active area of research. Stroke triggers neurogenesis in the DG [214], but the newly generated neurons produced post-stroke show an aberrant morphology [215,216]. The authors refer to this abnormal neurogenesis as responsible for the cognitive impairment found in mice after stroke and propose abolishing abnormal neurogenesis as a mechanism to improve cognitive performance post-stroke [40].

In this scenario, the activation of astrocytes in the SVZ and hippocampus after stroke results in the secretion of growth factors that promote the proliferation and differentiation of neural stem cells into new neurons. Faiz et al. have shown that SVZ-derived NSCs give rise to reactive astrocytes at the stroke site and can be converted to neurons in vivo following overexpression of Ascl1 [217]. This process is thought to be critical for the repair and recovery of the brain after a stroke. Reactive astrocytes also play an important role in forming new blood vessels after stroke, which is essential for the survival and integration of new neurons in the brain [218,219]. Several signaling pathways have been proposed to play roles in the adult neurogenic response to ischemia-induced stroke. The Notch signaling pathway regulates cell proliferation and differentiation during development and in adult tissue repair. In cooperation with ciliary neurotrophic factor (CNTF), Notch signals guide neural stem cells to generate astrocytes. Notch-CBF1 knockdown results in the conversion of neural stem cells to intermediate progenitor cells, which then generate neurons [220]. Studies have found that after a stroke, the Notch pathway assists the proliferation and differentiation of NSCs in the SVZ and hippocampus, leading to the formation of new neurons [221,222,223,224]. The sonic hedgehog (Shh) signaling pathway also plays a crucial role in early CNS development, influencing oligodendrocyte development [225]. After a cerebral ischemic stroke, Shh signaling improves the recovery of neurological function by increasing angiogenesis, neurogenesis, and oligodendrogenesis and reducing astrogliosis [226]. The upregulation of Shh expression and its transcription factor Gli1 in NPCs improved motor function in stroke mice, indicating its protective role in stroke [227,228].

Stroke-induced neurogenesis has been found to occur in humans, although the process is more limited compared to animal studies. Jin et al. found an increase in the number of new neurons in the hippocampus during the initial week post-stroke; however, this number returned to pre-stroke levels within 2–3 months [98]. An examination of brain tissue from advanced-age humans who have experienced ischemic stroke reveals an increase in the proliferation of SVZ cells and neuroblasts [229]. It is noteworthy that the mechanisms and extent of neurogenesis in stroke patients are not yet fully comprehended, and ongoing studies continue to investigate this process in humans.

## 8. Neurogenesis in Traumatic Brain Injury

Traumatic brain injury (TBI) can be defined in a number of ways. According to the WHO, a TBI is “…an acute brain injury resulting from mechanical energy to the head from external physical forces”. However, this definition excludes other causes such as “drugs, alcohol, medications, other injuries or treatment for other injuries, other problems, or penetrating craniocerebral injury” [230]. On the other hand, the National Institute of Neurological Disorders and Stroke gives the following definition: “TBI is defined as an alteration in brain function, or other evidence of brain pathology, caused by an external force.” This definition suggests that a greater context must be taken into account when considering what we acknowledge as TBI [231]. TBI accounts for 30–40% of all injury-related deaths and remains one of the leading causes of death and disability worldwide, with estimates of more than 50 million people suffering from TBI yearly [232]. TBI can result in long-term cognitive deficits and might be a major risk factor for late neurodegenerative disorders such as dementia and PD, reinforcing the view that TBI can evolve into a progressive lifelong illness [233,234,235]. TBI can be classified according to its severity, from a mild TBI (in which we can include concussions) to a moderate case or a severe TBI in the most serious of cases. Severe TBI is the one with the highest mortality rate, estimated, according to some studies, between 30% and 40% [236]. Pathophysiologically, two stages can be distinguished: the primary damage, inflicted at the time of injury and considered largely irreversible, and the secondary damage, which evolves over hours, days, weeks, months, or even over the patient’s lifetime. Secondary damage is caused primarily by the body’s response to primary damage and is responsible for the extensive tissue loss seen in TBIs. This secondary injury, which remains the target of most treatment strategies, is mediated by several factors, including excitotoxicity, neuroinflammation, mitochondrial dysfunction, oxidative stress, axonal degeneration, and apoptosis. There is numerous evidence showing the activation of various CNS progenitor pools with potential regenerative capabilities. A better understanding of this progenitor response is crucial for the development of new treatments and new neuroregenerative strategies [237].

Regarding the neurogenic process, studies have shown that TBI induces an upregulation of neurogenesis in various types of TBI models [238]. When a TBI occurs, both SVZ and SGZ neurogenic niches react by activating NSCs, stimulating the proliferation and differentiation of NSCs into neuroblasts, and altering the migration patterns of these neuroblasts to lead them towards the injured area (Figure 1F) [239,240,241]. However, only a very small number of newly born neurons reach the lesion [62,242]. This is a consequence of the release of inflammatory signals that create a gliogenic/non-neurogenic environment within the injured tissue [243,244]. The injury-induced adult neurons are also capable of functional integration into the hippocampal network [245] and are directly associated with spontaneous cognitive and functional recovery observed following injury [238,246,247]. However, this injury-enhanced cell proliferation is relatively transient and is only observed during the first week post-injury [248]. It has been shown that as these neurons migrate long distances through adult brain tissues, they are supported by various guidance signals such as BDNF [249,250]. BDNF and its receptors play a very important role when it comes to guiding neuronal migration. However, when a TBI occurs, the role of BDNF and how it affects the migration of neuroblasts is still not very clear [251,252].

Recently in 2023, Wu et al. have shown that after eliciting a TBI by performing a controlled cortical impact, the migration of neuroblasts begins the day after the lesion is produced, reaches a peak at day 7, and remains until day 21. These neuroblasts seem to migrate along the activated astrocytic tunnel and are directed by the BDNF gradient between the SVZ and injured cortex after TBI [90].

Neurogenesis has traditionally been thought to be restricted to the aforementioned neurogenic niches [253]. However, since in cases of TBI the greatest neuronal death occurs in the cortex, cortical neurogenesis is of greater interest. Braun et al., used a model of cortical contusion trauma in adult rats in which they demonstrated that there are cells with a high expression of βIII-tubulin within the site of cortical injury at 7 days post-injury, indicating that neurogenesis is not confined to the SVZ/hippocampus following injury [254]. In later studies, researchers isolated tissue from the cortical lesion site in adult rats and managed to grow neurospheres in vitro, which were capable of generating neurons (Tuj1 + cells), astrocytes (GFAP + cells), and oligodendrocytes (O4 + cells) [255]. Despite promising evidence of cortical, hippocampal, and SVZ neurogenesis post-injury, the longer-term survival and functional integration of newly born neurons remained unexplored until recently. Several studies have demonstrated aberrant dendritic branching and migration patterns of newborn neurons within the hippocampus post-injury [253].

## 9. Strategies to Promote Neurogenesis in the Damaged Brain Regions

Neuronal replacement strategies for the brain include transplantation of exogenous cells from a neuronal lineage or potentiation of endogenous neurogenesis. Several groups worldwide have tried to facilitate neuronal replacement by transplanting stem cells into the damaged brain. Different sources of donor cells are used for transplantation into the injured or diseased area, including fetal neurons, embryonic NCS-derived, embryonic stem cell (eNSC)-derived, and induced pluripotent stem cell (iPSC)-derived neurons. These studies have proven to achieve both clinically and experimentally remarkable and meaningful outcomes, demonstrating that neuronal replacement is feasible and achieves behavioral recovery in several paradigms. In addition to having the advantage that exogenous cells can be transplanted into any damaged region. However, the best results have been obtained with fetal neurons, which entails ethical implications, and the pool of neurons is more restricted [256,257]. Potentiating endogenous neurogenesis is the other strategy currently being studied to regenerate brain tissue. Four important stages of the neurogenic process can be modulated in order to promote endogenous neurogenesis and facilitate neuronal replacement: (i) potentiating NSCs activation and proliferation within the lesion [27], (ii) leading the fate of these progenitors into a neuronal phenotype [162], (iii) transforming glial cells into NSCs by the use of factors such as NeuroD1 [258] or SOX2 [259], and (iv) generating an environment that favors neurogenesis in neurogenic regions and the migration of neuroblasts towards the damaged area, as well as the survival, differentiation, and posterior integration of the newly generated neurons into existing circuits.

**Figure 2:**
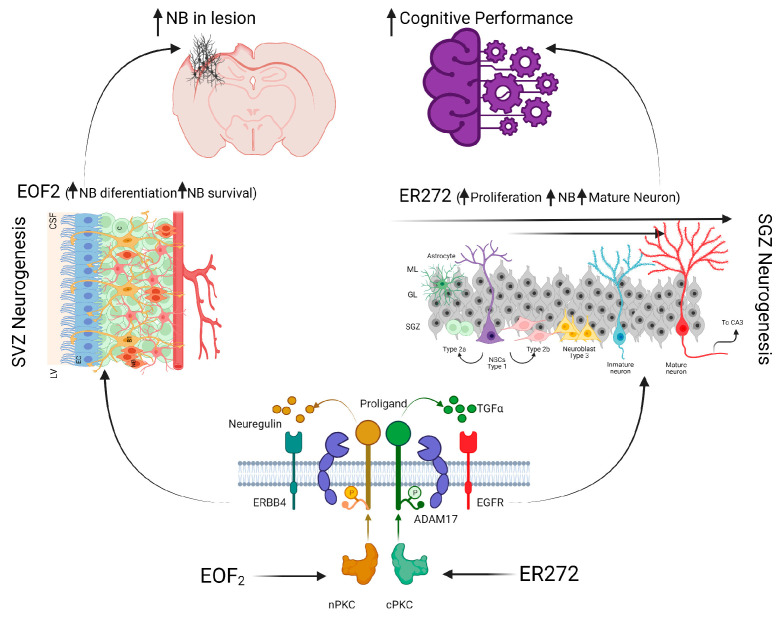
Effects of diterpenes on adult neurogenesis and its functional implications. In the bottom, it is represented how the different diterpenes act on their kinase targets, initiating the PKC-ADAM17 signaling pathways. In the top is represented its effects on SVZ and hippocampal neurogenesis and its potential benefits. EOF2 activates nPKC, which mainly induces neuregulin release and promotes neuroblast differentiation and migration to the injured areas. ER272 activates cPKC, which induces TFGα release and active hippocampal neurogenesis, improving cognitive performance. ADAM17, tumor necrosis factor-α-converting enzyme; CSF, cerebrospinal fluid; EC, ependymal cells; EGFR, epidermal growth factor receptor 1; GL, granular layer; ML, molecular layer; LV, lateral ventricle; NB, neuroblast; NSCs, neural stem cells; cPKC, classical protein kinase C; nPKC, novel protein kinase C; SVZ, subventricular zone; SGZ, subgranular zone; TFGα, transforming growth factor alpha. Created with BioRender.

The generation of neurons from endogenous NSCs to achieve the replacement of death neurons in an injured or diseased CNS region requires the fine tuning of the molecular micro-environment to generate a neurogenic niche in which endogenous NSCs could differentiate into functional and mature neurons and in which migration is facilitated. Several molecules are involved in these processes and may constitute targets for pharmacological compounds. Different endogenous growth factors and chemokines-related molecules have been administered via intracerebral injections to damaged/injured brains and have proven to promote neurogenesis and/or migration. It is the case of the growth factors BDNF [260,261,262], IGF-1 [263], GDNF [264], epidermic growth factor (EGF) [265], TGF-α [239], or vascular endothelial growth factor (VEGF) [266] and chemokine-related molecules such as the CC chemokine ligand 2 (CCL2) [90] or the CXCL2 N-terminal end [267]. Likewise, other neurogenic signaling molecules, such as noggin [268], have been administered through intraventricular administration, resulting in a significant increase in hippocampal neurogenesis and migration in APP/PS1 mice [269]. Although other endogenous and synthetic compounds have been studied via intraperitoneal or intravenous injections [270,271,272,273], yielding promising results, the search for small molecules capable of penetrating the blood-brain barrier and thus being optimum for the development of non-invasive therapies is one of the challenges of current research. Although still in the early stages of development, this field is giving optimistic and encouraging results that should be further investigated. Till today, the intranasal route seems to be the most feasible non-invasive method of administration. This approach has been explored by different groups [274,275], including ours [244,276]. In this regard, our group has described two diterpenes capable of promoting neuroblast differentiation and migration from the SVZ towards a controlled TBI (EOF2, CAS number 2230806-06-9) [244] or of activating hippocampal neurogenesis and improving cognitive performance (ER272) [276]. In brain injuries, the metalloproteinase ADAM17 releases the transforming growth factor alpha (TFGα) which activates the epidermal growth factor receptor (EGFR), thus promoting the creation of a gliogenic/non-neurogenic environment [242]. Both molecules are overexpressed in the injured area, and specific inhibition of ADAM17 has been demonstrated to facilitate neurogenesis, migration, and survival of newly formed progenitors and neuroblasts [62]. Interestingly, different isoenzymes of the protein kinase C (PKC) family can regulate ADAM17 activity, thus contributing to the neurogenicity of the injury environment [277,278]. In this sense, our group described that both EOF2 and ER272 are able to activate different PKCs [279,280]. Specifically, EOF2 activates novel PKCs [244], thus facilitating the release of neuregulin via ADAM17, whereas ER272 activates classical PKCs and facilitates the release of TGF-α [276], as described in Figure 2. Moreover, in recent work, it has been demonstrated that ER272 prevents the neurogenic alterations and cognitive impairment found in the aging mouse model SAMP8 that are present characteristics of AD [281]. Therefore, compounds of this kind represent a promising pharmacological tool to promote neurogenesis and regeneration of the damaged brain.

## 10. Conclusions

In conclusion, neurogenesis and migration in both the SVZ and DG are altered in several neurological disorders. Alterations found in the DG and SVZ niches differ depending on the insult. However, all these reports suggest that regeneration of damaged brain regions may be achieved by developing strategies aimed at promoting endogenous neurogenesis. In this sense, the discovery of target molecules whose activities can be pharmacologically modulated is crucial. Targets to be used to develop pharmacological agents vary from EGFR inhibitors, PKC activating diterpenes, Noggin, or BDNF, although other molecules may arise in the near future after further studies are performed to elucidate the mechanisms underlying neurogenesis and neuroblast migration toward brain injuries.

## Figures and Tables

**Table 1:** Molecules involved in neuroblast migration.

Molecule	Brain Location	Secretor	Receptor	Role	References
Prokineticin-2 (PK2)	OB	Granular and periglomerular layers of the OB	PRK1/PRK2	Chemoattractant	Ng et al., 2005 [78].
Netrin-1	OB and RMS	Mitral cells (OB) and astrocytes (RMS)	Deleted in colorectal cancer (DCC)	Chemoattractant	Murase and Horwitz., 2002 [79].
Glial cell line-derived neurotrophic factor (GDNF)	OB and RMS	OB cells	NCAM	Chemoattractant	Paratcha et al., 2006 [80].
Hepatocyte growth factor (HGF)	SVZ and RMS	Astrocytes	Met	Chemoattractant (restricts cells to the RMS)	Garzotto et al., 2008 [81]; Wang et al., 2001 [82].
Monocyte chemoattractant protein-1 (MCP-1)	Injury (focal ischemia)	Injury-activated microglia and astrocytes	CCR2	Chemoattractant	Yan et al., 2007 [84].
Osteopontin (OPN)	Injury (focal ischemia)	Activated microglia and macrophages	Beta1-integrins	Chemoattractant	Yan et al., 2009 [83].
Slit1	Septum, RMS and SVZ	Type A and C cells, migrating neuroblast (SVZ, RMS)	Robo2, Robo3/Rig-1	Repellent	Nguyen-Ba-Charvet et al., 2004 [86].
	RMS	Migrating neurons	Robo2 (astrocytes)	Modifies glial tube thus promoting migration	Kaneko et al., 2010 [67].
	Injured striatum (stroke)	Migrating neurons	Robo2 (astrocytes)	Promotes migration through astrocytes	Kaneko et al., 2018 [73].
Slit2	Septum	Undefined	Robo2, Robo3/Rig-1	Repellent	Nguyen-Ba-Charvet et al., 2004 [86].
	CSF of LV, creating a gradient with lower concentration at rostral position	Choroid plexus	Robo2	Repellent	Sawamoto et al., 2006 [89].
Insulin growth factor 1 (IGF-1)	SVZ	Choroid plexus	IGF-IR	Repellent	Hurtado-Chong et al., 2009 [87].
GABA	Anterior SVZ and RMS	Neuroblast	GABA_A_R	Reduce migration rate	Bolteus and Bordey, 2004 [88].
Vascular endothelial growth factor (VEGF)	SVZ and RMS	Glial cells	VEGFR-2	Migration along blood vessels	Wittko et al., 2009 [71]; Bozoyan et al., 2012 [72].
Stromal-derived factor 1 (SDF-1)	Injury (stroke)	Stroke-activated cerebral endothelial cells	CXCR4	Migration along blood vessels	Ohab et al., 2006 [74]; Robin et al., 2006 [75].
	Injury (stroke)	Reactive astrocytes and activates microglia	CXCR4	Migration along blood vessels	Thored et al., 2006 [76].
Angiopoietin 1 (Ang1)	Injury (stroke)	Stroke-activated cerebral endothelial cells	Tie2	Migration along blood vessels	Ohab et al., 2006 [74].
Brain-derived neurotrophic factor (BDNF)	OB, RMS	Vascular endothelial cells	p75NTR	Migration along blood vessels	Snapyan et al., 2009 [68].
	Injury (stroke)	Endothelial cells	p75NTR	Migration along blood vessels	Grade et al., 2013 [77].
	Injury (TBI)	Reactive astrocytes	-	Chemoattractant	Wu et al., 2023 [90].
Polyasilated form of neural cell adhesion molecule (PSA-NCAM)	SVZ and RMS	Migrating neuroblast	NCAM	Migration by creating neuroblast chains, maintenance of RMS structure	Battista and Rutishauer., 2010 [91].
Laminin	RMS	ECM	Beta1-integrins	Migration by creating neuroblast chains	Belvindrah et al., 2007 [64].
	Blood vessels to injury (stroke)	ECM	Beta1-integrins	Migration by creating neuroblast chains along blood vessels	Fujioka et al., 2017 [59].
Neuregulin 1 (NRG1)	RMS	Undefined	ErbB4	Permissive guidance cue, chemotropic agent or mitogen	Anton et al., 2004 [65].
	SVZ	Neuroblast	ErbB4	Chemoattractant/Prevents neuroblast migration toward OB leading cells toward the source of NRG1	Ghashghaei et al., 2005 [66].
Neuregulin 2 (NRG2)	SVZ	Neuroblast	ErbB4	Chemoattractant. Attracts neuroblasts to the OB	Ghashghaei et al., 2005 [66].

The table summarized the role of some of the molecules involved in the migration of neuroblasts in the adult brain, either under physiological conditions or after brain injury, as reflected in the column “brain location”. Note that controversial or contradictory information for a single molecule is reported in different sub-rows for that molecule. ECM, extracellular matrix; OB, olfactory bulb; RMS, rostral migratory stream; SVZ, subventricular zone; TBI, traumatic brain injury.
